# Transarterial fiducial marker implantation for CyberKnife radiotherapy to treat pancreatic cancer: an experience with 14 cases

**DOI:** 10.1007/s11604-020-01040-1

**Published:** 2020-09-11

**Authors:** Akira Imaizumi, Takuji Araki, Hiroki Okada, Yu Sasaki, Takafumi Komiyama, Toshihiro Suzuki, Hiroshi Takahashi, Hiroshi Onishi

**Affiliations:** 1 Department of Radiology, Yamanashi University, 1110 Shimokato, Chuo, Yamanashi 409-3898 Japan; 2 Kasugai CyberKnife Rehabilitation Hospital, 436 Kokufu, Kasugai-cho, Fuefuki, Yamanashi 406-0014 Japan

**Keywords:** Transarterial fiducial marker implantation, Pancreatic cancer, CyberKnife radiotherapy, Real-time tumor tracking

## Abstract

**Purpose:**

The purpose of this study was to evaluate the safety and feasibility of transarterial fiducial marker implantation for CyberKnife radiotherapy to treat locally advanced pancreatic cancer.

**Materials and methods:**

Fifteen pancreatic cancer patients were enrolled for transarterial marker implantation. Embolization platinum coils were implanted as a fiducial marker within 20 mm of the cancer edge, and preferably within 3 mm. The technical success of the implantation was defined as implantation of at least one fiducial marker within 20 mm of the target tumor. Irradiation was performed using the CyberKnife system.

**Results:**

For 14 of 15 patients, transarterial implantation was successfully performed, and for 13 of 14 patients, the tracking marker was implanted within 3 mm of the cancer. Tracking instability was observed in two patients, but irradiation was accomplished in all 14 patients. No major complications caused by the implantation procedure were observed. The median overall survival after irradiation was 13.8 months, and the 1- and 2-years survival rates were 62.9% and 32.3%, respectively.

**Conclusion:**

Transarterial fiducial marker implantation for pancreatic cancer can be safely performed for tracking, and it will be a valuable alternative approach to percutaneous fiducial marker implantation.

## Introduction

Previous studies have shown that CyberKnife radiotherapy is effective in patients with locally advanced pancreatic cancer [1]. This therapy decreases delays in the systemic treatment of pancreatic cancer while increasing local tumor control. Local tumor control improves patient quality of life, by controlling pain and decreasing the occurrence of gastric obstruction or duodenal obstruction [2]. Further, local control of tumors achieved through CyberKnife radiotherapy prevents distant metastasis [2]. Real-time tumor tracking during CyberKnife radiotherapy is currently used in the treatment of lungs, abdominal, and pelvic tumors, which have respiratory movement. The pancreas is an organ that has respiratory movement [3]. Therefore, to perform CyberKnife radiotherapy for pancreatic cancer, real-time tumor tracking is needed. For implantation of fiducial marker to real-time tracking, computed tomography (CT) or ultrasound (US)-guided percutaneous implantation is commonly used [4, 5]. However, the percutaneous approach is difficult to perform when other organs are located between the puncture point and the target. To overcome this, we performed transarterial fiducial marker implantation by microcatheter, guided by angiography. In this study, we evaluated the technical and clinical outcomes of transarterial marker implantation for CyberKnife radiotherapy in pancreatic cancer.

## Materials and methods

### Patients

**Fig. 1:**
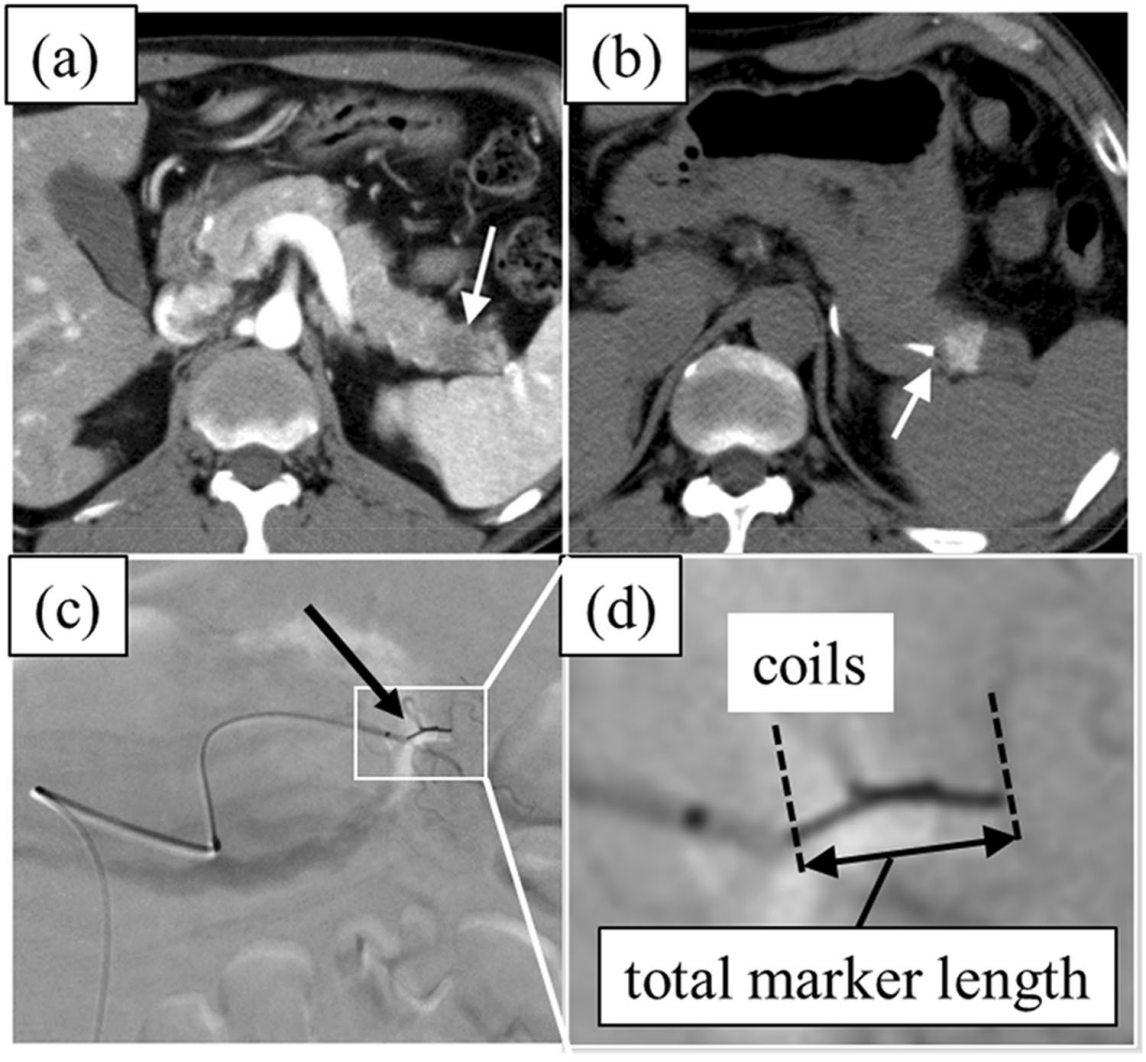
An example case of transarterial marker implantation for pancreatic tail cancer. **a** Pre-procedural dynamic contrast-enhanced (CE) CT was performed to reveal the tumor location (marked by white arrow). **b** A microcatheter is inserted into the artery branch near the target cancer and angio-CT is performed to determine the location of the microcatheter tip (white arrow) relative to the target cancer. Residual contrast media injected in the previous angiography also appears on this image. **c**, **d** Post-procedural angiogram of the implanted fiducial marker (black arrow; 2 pieces of 5-mm-long straight embolization microcoils with a 0.018-inch diameter)

Between September 2012 and December 2016, 17 patients were diagnosed with pancreatic cancer and judged as unable to undergo surgical resection due to local progression, distant metastasis, or advanced age. They were referred to our hospital for CyberKnife therapy. Fiducial marker implantation was required to perform CyberKnife therapy for real-time tumor tracking. Radiation oncologists and interventional radiologists assessed each case by pre-procedural imaging studies (CT, magnetic resonance imaging (MRI) (Fig. 1a), and judged that in 15 of 17 patients, percutaneous marker implantation was too difficult to perform due to a patient’s anatomical features and/or target lesion location (another organ located between the tumor and puncture sight, or the target was too far from the skin). The characteristics of the 15 patients included in this study are shown in Table 1. Eleven of 15 patients had a history of systemic chemotherapy before and/or after Cyberknife therapy. The other four patients had no history of chemotherapy during the progress. The institutional review board of our hospital approved the study protocol, and all patients gave written informed consent for the research protocol and the procedure.

**Table 1:** Patient characteristics (*n* = 15)

Characteristics	Value
Sex (male: female)	10:5
Age (years)	
Mean (range)	70.6 (41–88)
Local progression^a^ (T2:T3:T4:recurrence)	2:2:8:3
Tumor location in pancreas (head:body:tail)	7:6:2
Lymph node metastasis^a^ (N0:N1 and above)	11:4
Distal metastasis^a^ (M0:M1)	12:3
History of chemotherapy (yes/no)	11:4^c^
Chemotherapy before RT	7^b^
GEM + S-1	1
S-1 only	4
GEM only	1
nab-PTX + GEM	1
Chemotherapy after RT	5^b^
nab-PTX + GEM + nivolumab	1
nab-PTX + GEM	1
GEM only	1
S-1 only	2
RT dose (Gy)	
Median (range)	50 (40–60)
RT fraction	
Median (range)	10 (5–15)

*RT* radiotherapy, *GEM* gemcitabine, *nab-PTX* nab-paclitaxel

^a^UICC TNM Classification of Malignant Tumors, 8th edition

^b^An overlap existed before and after RT

### Fiducial marker implantation

All implantation procedures were performed using an angio-CT unit (INFX 8000C/JU and Aquilion LB, CANON Medical Systems, Tochigi, Japan). Marker implantation was performed using the 3-Fr catheter system, which included a 3-Fr 25 cm sheath, a 3-Fr Shepard Hook type catheter, and a 2.0/2.4 Fr microcatheter (Carnerian Marvel, Tokai Medical Products, Gifu, Japan). An embolization platinum microcoil with a 0.018-inch diameter (Hilal®, Tornado® Embolization Microcoil (Cook Medical, Indiana, USA)) was implanted as a fiducial marker. We chose the size and shape of coils by the target vessel size. If the target vessel was thin, the straight coil seemed to be appropriate. On the other hand, when the target vessel was a little bit thick, we chose the single-curl or multi-curl coil, because curled coils had stronger radial force than straight type and had lower risk of distal migration. First, digital subtraction angiography (DSA) was performed on the superior mesenteric artery (SMA) and the celiac artery (CA) to determine the arterial anatomy. Second, the artery branch nearest to the target tumor was identified and the microcatheter was inserted into the artery branch. Third, plain CT was performed to confirm that the tip of the microcatheter was located near the target lesion. If the relationship between catheter tip and target tumor was still unclear on this plain CT, selective contrast-enhanced CT was performed. Then, one or two pieces of embolization coil were implanted in parallel by position (Fig. 1b); they formed one group of markers. The intended implantation position was within 20 mm of the cancer edge, and preferably within 3 mm. One or two groups of markers were implanted; in most cases, irradiation was available with only one marker. However, in our earlier cases, another marker was implanted as a backup. Post-procedural CT was performed to determine if the coil was correctly implanted near the cancer edge, and if so, the implantation procedure was completed (Fig. 1c). All implantation procedures were performed within 2 or 3 days of hospitalization to watch the short-time complications. If there was no bleeding at the puncture site or other apparent symptoms after implantation and blood test results (blood count, C-reactive protein, serum amylase level) were within the normal range, the patient was discharged and went to irradiation.

### Radiation therapy

For all patients, the Synchrony® Respiratory Tracking System (Accuray, Sunnyvale, California, USA) was used for real-time tracking the tumors. To create treatment plans, a four-dimensional treatment planning system (Multiplan version 5.2; Accuray, Sunnyvale, California, USA) was used, and the CyberKnife G4 radiosurgery system (Accuray, Sunnyvale, California, USA) was used for treatment. Patients were simulated in a supine position and immobilized with a vacuum cushion. Contrast-enhanced dynamic CT images were used to identify the gross tumor volume (GTV). The clinical target volume (CTV) was equal to the GTV. Internal target volume (ITV) was obtained as the fiducial fusion of CTV in inspiratory and expiratory phases. Safety margins around the tumor of 3 mm in the lateral and vertical directions and 3.75 mm in cranio-caudal direction were added to the ITV, and this was defined as planning target volume (PTV). Dose constraints for stereotactic body radiation therapy for pancreatic cancer in our institution are listed in Table 2. The α/β ratio of the biological equivalent dose was 3 Gy. A total median dose of 50 Gy (range 40–60 Gy) was delivered in 10 fractions (median, range 5–15) to the PTV D95%. During treatment, respiratory tumor motion was actively compensated for by the dynamic Synchrony® Respiratory Tracking System.

**Table 2:** Dose constraints

Location	D1cc (Gy)	D10cc (Gy)	Dmax (Gy)
Stomach			
Duodenum	< 144 Gy	< 105 Gy	–
Small intestine			
Esophagus	< 160 Gy	< 120 Gy	–
Trachea/bronchus			
Heart	< 175 Gy	< 140 Gy	–
Large vessel			
Spinal cord	–	–	< 75 Gy

*D1cc* dose delivered to 1% of the target volume, *D10cc* dose delivered to 10% of the target volume, *Dmax* maximum dose

### Follow-up assessment

Technical success of implantation was defined as implantation of at least one fiducial marker within 20 mm of the target tumor. Other recorded features used to assess success included the target artery, coil character, number of implanted coils, distance from coil to target, total marker length (measured using the MPR image of the post-procedural plain CT), number of markers, procedure duration, number of plain/contrast-enhanced CTs during the procedure, availability of feather stable tracking (yes/no), accomplishment of radiotherapy (RT) (yes/no), complications caused by the marker implantation procedure, and toxicity by RT (graded according to the NCI Common Terminology Criteria for Adverse Events, version 4.03). The total marker length was defined as the maximum diameter of each marker group (Fig. 1d). Complication caused by the implantation was defined as any abdominal symptoms observed after implantation or any vessel injury that occurred during the angiography procedure. We asked patients to describe their symptoms during hospitalization, and radiation oncologists performed follow-up interviews when the outpatient visited for RT planning.

### Statistical analysis

Overall survival (OS) and local progression-free survival were determined after RT by generating survival curves by the Kaplan–Meier method. The median survival month and overall survival rate for 1 and 2 years were calculated from the Kaplan–Meier plots. All statistical analyses were performed with EZR (Saitama Medical Center, Jichi Medical University, Saitama, Japan), which is a graphical user interface for R (The R Foundation for Statistical Computing, Vienna, Austria). More precisely, it is a modified version of R commander, designed to add statistical functions frequently used in biostatistics [6].

## Results

**Fig. 2:**
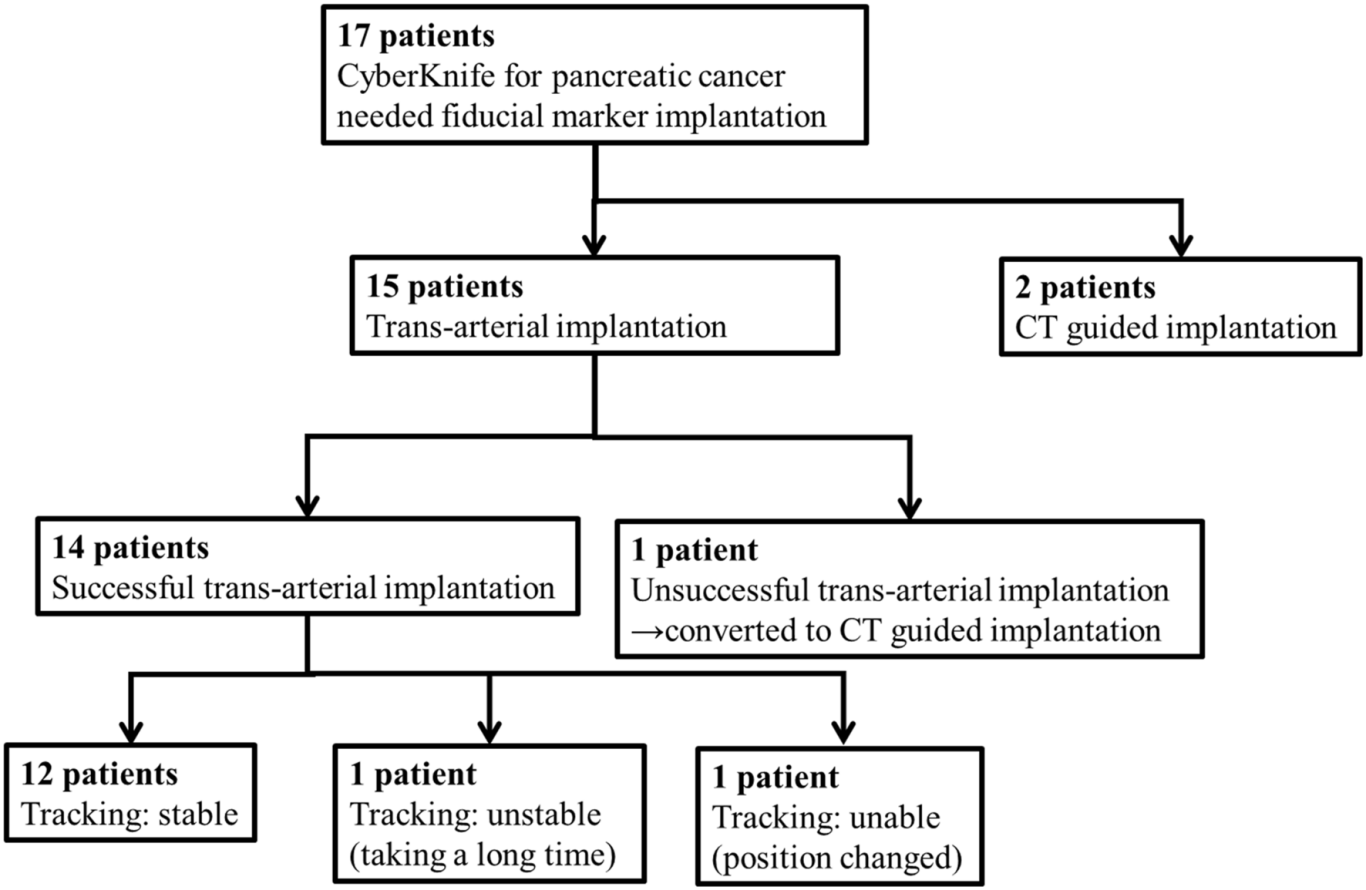
Kaplan–Meier estimates of overall survival and local progression-free survival after radiation therapy of all patients (*n* = 14)

Implantation and irradiation of each patient is diagrammed in Fig. 2 and summarized as follows. In 14 of 15 patients, transarterial fiducial marker implantation was successfully performed (a technical success rate of 93%). In 13 of 14 cases, the puncture site was the right femoral artery; in the one other patient, a left brachial approach was chosen since he had a history of aortic artery repair. Transcatheter implantation of fiducial markers failed for only 1 of 15 cases. This failure occurred because there was no appropriate artery located near the pancreatic cancer target. In this case, we performed CT again during the angiography procedure, which indicated that CT-guided percutaneous implantation was an available alternative, which we used for successful implantation.

**Fig. 3:**
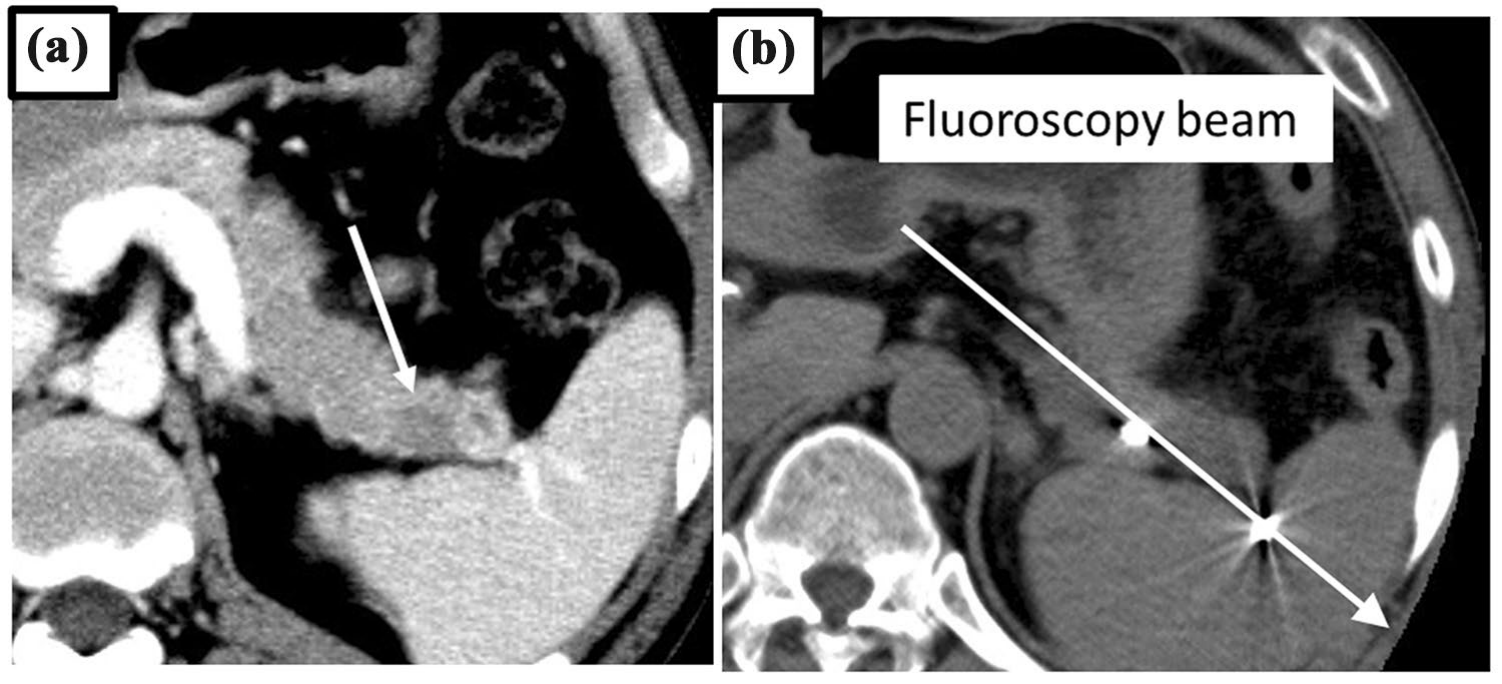
Summary of patient fiducial marker implantation and irradiation

**Fig. 4:**
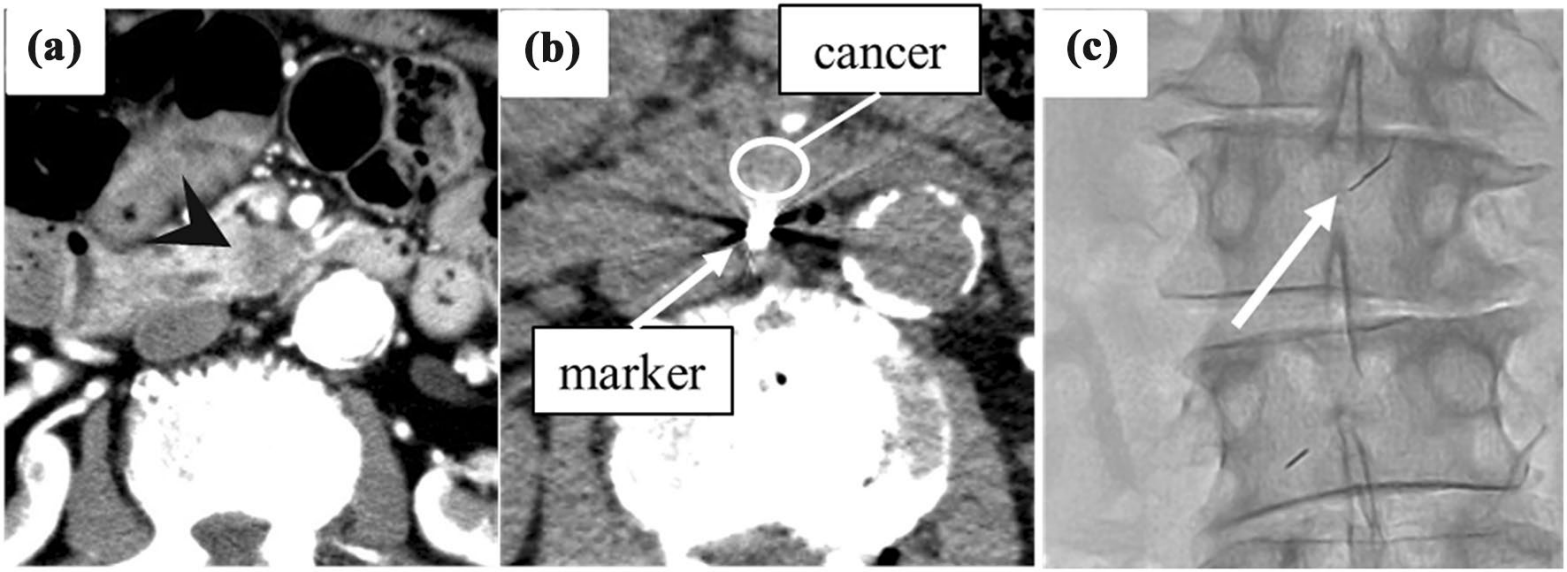
A case of tracking in parallel to the fluoroscopy beam. **a** Pre-procedural dynamic contrast-enhanced (CE) CT of the cancer target in the pancreas tail (white arrow). **b** Post-procedural plain CT shows two fiducial markers that were implanted on opposite ends of the cancer aligned with a fluoroscopy beam (white arrow), preventing the CyberKnife system from distinguishing the two separate markers

Selective contrast-enhanced CT was performed during the procedure in four cases; in the other 10 cases, marker implantation was accomplished with DSA and plain CT only. For 13 of 14 patients, the tracking marker was implanted within 3 mm of the cancer. Stable marker tracking was successful in 12 of 14 patients, as defined as the ability of the CyberKnife to distinguish the fiducial markers. In one case, each marker was implanted successfully but was lined up along a tracking fluoroscopy beam (Fig. 3). This meant that the CyberKnife could not distinctly recognize them as separate markers. Changing the patient’s position to the oblique supine position and adjusting the RT setup enabled the tracking system to work correctly. In a second case where implantation occurred successfully, two 10-mm-long straight coils were implanted around the pancreatic head cancer target (Fig. 4). Since a total marker length greater than 10 mm was too long for stable marker tracking, irradiation took longer but was completed without additional marker implantation. Migration of the fiducial markers was not observed in the 14 cases.

**Fig. 5:**
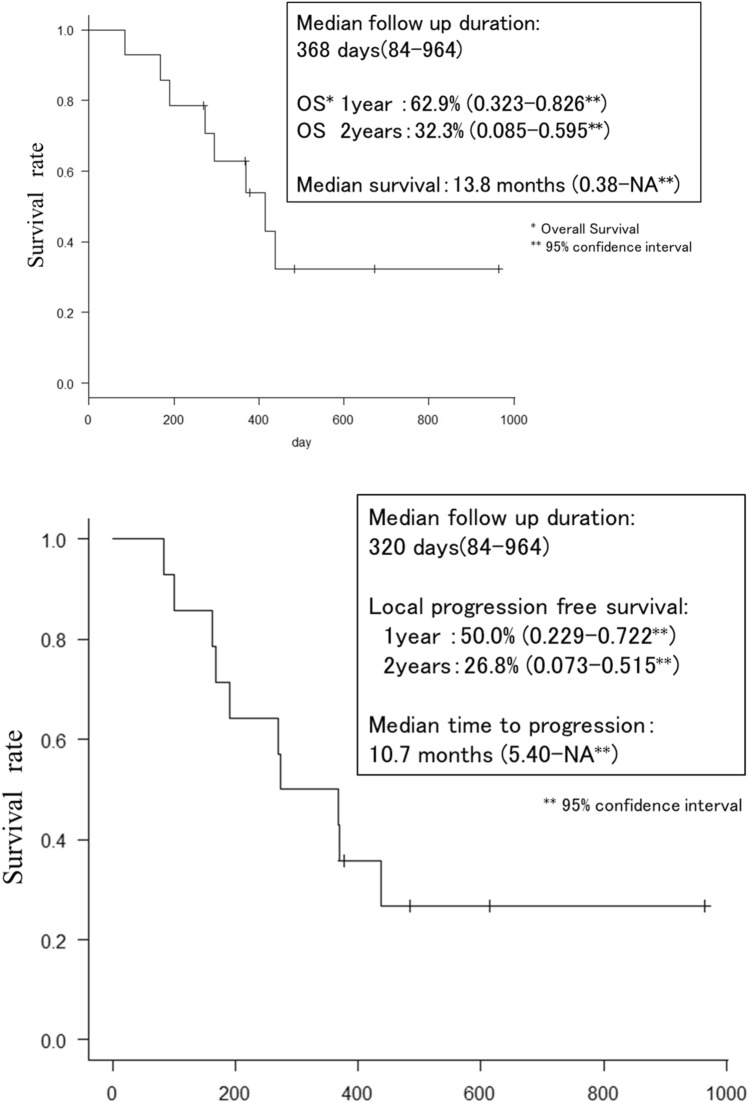
A case of unstable tracking. **a** Pre-procedural dynamic contrast-enhanced (CE) CT of the cancer target in the pancreas head (black arrow head). **b** Two straight-shaped coils 10 mm in length were implanted nearby the cancer. **c** The post-implantation fluoroscopy image shows that the total marker length was too long for stable tracking

Ultimately, in all 14 cases in which the marker was implanted by the transarterial procedure, irradiation was accomplished. Complications caused by the implantation procedure were not observed in any patient. Toxicity caused by RT was observed in two cases; one was a grade 3 gastric hemorrhage and the second was a grade 2 duodenal ulcer. In both cases, the target tumor location was the pancreas head. The grade 2 patient was treated with medication. The grade 3 patient underwent endoscopic hemostasis, which failed, and then trans-arterial embolization. The summary of all 15 cases is presented in Table 3. The median follow-up period after the day of the beginning of RT was 368 days (range 84–964 days). The median overall survival after RT was 13.8 months (95% confidence interval (CI): 4.67–N/A). The 1-year and 2-year overall survival rates were 62.9% (95% CI = 0.323–0.826) and 32.3% (95% CI = 0.085–0.596), respectively (Fig. 5). The median time to local progression was 10.7 months (95% CI = 5.4–N/A). The 1-year and 2-year local progression-free survival rates were 50.0% (95% CI = 0.229–0.722) and 26.8% (0.073–0.515), respectively.

**Table 3:** Patient features

Patient	Location	Target artery	No. of marker group	Coil character^a^	Stable tracking	Distance to tumor	Total marker length	Procedure duration (minute)	Number of Plain CT	Number of Selective CE-CT
1	Head	ASPD, DPA	1	2-2HILAL	Ok	< 3 mm	4 mm	89	0	3
2	Body	Convert to percutaneous implantation								
3	Body	LGA, MCA	2	C3 mm + 5 mm, 2-2HILAL	Ok	< 3 mm, 14 mm	6 mm/7 mm	216	3	0
4	Body	DPA, LGA	2	2-2HILALx2,7 mm	Ok	< 3 mm, 13 mm	5 mm/8 mm	72	2	1
5	Head	PSPD, MCA	2	2-2HILAL, 2-2HILAL	Ok	< 3 mm	5 mm/5 mm	140	3	1
6	Tail	PMA, SpA	2	5 mm, 2-2HILAL	**Unable**	7 mm, 9 mm	7 mm/7 mm	228	3	2
7	Head	PSPD, ASPD	2	2-2HILAL, C3 mm	Ok	< 3 mm, 10 mm	4 mm/6 mm	113	3	0
8	Head	IPD, PSPD	2	5 mm × 2 5 mm × 3	Ok	< 3 mm	6 mm/7 mm	114	3	0
9	Head	PSPD	1	2-2HILAL	Ok	< 3 mm	5 mm	118	3	0
10	Body	DPA, LGA	2	2–4, 2–4	Ok	< 3 mm	7 mm/8 mm	63	2	0
11	Tail	DPA	1	C3 mm	Ok	< 3 mm	7 mm	78	1	0
12	Head	IPD	1	10 mm × 2	**Unstable**	< 3 mm	13 mm	106	1	0
13	Body	DPA	1	C3 mm, 2-2HILAL	Ok	< 3 mm	6 mm	67	1	0
14	Body	DPA	1	5 mm × 2	Ok	< 3 mm	6 mm	139	1	0
15	Head	DPA	1	2-2HILAL	Ok	< 3 mm	5 mm	45	1	0

*DPA* dorsal pancreatic artery, *ASPD* anterior superior pancreaticoduodenal artery, *PSPD* posterior superior pancreaticoduodenal artery, *IPD* inferior pancreaticoduodenal artery, *SpA* splenic artery, *PMA* pancreatic magna artery, *MCA* medial colic artery, *LGA* left gastric artery, *2-2HILAL* 2 mm/2 mm-diameter helical-shaped coil, *C3mm* 3 mm-diameter C-shaped coil, *5 mm/7 mm/10 mm* straight coil, *2–4* 2 mm/4 mm-diameter tornado-shaped coil

## Discussion

Here, transarterial fiducial marker implantation for CyberKnife radiotherapy was successful in fourteen cases to treat locally advanced pancreatic cancer, demonstrating that this technique is an effective alternative to the percutaneous approach. In the case where tracking was unable to occur, the target cancer was located on the pancreas tail and the two markers were implanted on opposite ends, in parallel to the fluoroscopy beam, which prevented recognition by CyberKnife tracking. Tracking was enabled only after adjusting the patient’s position during RT setup. The incident angle of the CyberKnife tracking system’s fluoroscopy beam is about 45° to the horizontal line; from this case, it is clear that the markers should not be implanted parallel to this 45° line.

In the case of unstable tracking, fiducial marker tracking by the CyberKnife system would sometimes stop. In this case, 2 pieces of 10-mm-long straight coils were implanted in series in the same vessel, and the maximum length of the combined markers was over 10 mm. In another case without tracking errors that we had experienced, the total length of the tracking marker was shorter than 8 mm. From that instance, we considered that too-long marker lengths lead to unstable tracking. Before this case, we had considered that too-small and short fiducial markers might have insufficient visibility for stable tracking because of prior experience with unstable tracking using a single 5-mm coil (unpublished). We had thought that the total size of the marker should be larger and longer. A phantom simulation experiment of our institution suggested that the marker tracking might become unstable when the maximum length of the markers was over 8 mm. These factors lead to our consideration that a coil that was too small and short might have insufficient visibility for stable tracking; however, a marker that is too long and large is also problematic. Total marker length should be shorter than 8 mm in future procedures, to ensure that stable tracking occurs.

Especially, in later cases, we performed the marker implantation without CT angiography. In these cases, we could find the adjacent vessel to the marker implantation using DSA and plain CT only. It was up to the operator in each case to decide whether to use CT angiography or not. However, in some cases, we took too much time to accomplish the implantation. According to the retrospective review of each procedure, much of the entire procedure time was spent searching for the adjacent vessel. Although CT angiography requires considerable contrast agents and a bit of time, it might provide information about the local anatomy and might reduce the total procedure time. There could be room for improvement regarding our method. Although not available in our institution, 3D vessel tracking software might be helpful to search for the adjacent vessel.

Before performing this transarterial implantation, we expected that complications due to implantation were local ischemic reactions caused by the embolizing function of the implanted coil, but no complications caused by the implantation procedure were observed. We reason that the two small pieces of coil have limited embolization power, and pancreatic tissue has a rich arterial network and collateral pathway [7], so the local ischemic reaction was likely limited and thus caused no clinical symptoms. Although it was reported that the risk of severe complications caused by a percutaneous approach is not very high, local hemorrhage and fiducial marker migration might occur [4]. One beneficial feature of transarterial implantation is that the needle used to insert the marker does not penetrate the tissue between the puncture site and the target organ. This potentially causes less injury to other organs in the penetration tract and may reduce the risk of tumor dissemination and marker migration. In addition to CT or US-guided percutaneous approaches, endoscopic ultrasound (EUS)-guided trans-gastrointestinal marker implantation has been reported [8–10] as a successful technique. This is also good alternative technique, but there is not dedicated product for EUS-guided marker implantation, and it also requires the off-label fiducial marker use. Another characteristic of EUS-guided approach is that the marker implantation might be more difficult when the target locates on pancreatic tail. Based on the success of these proceeds, as well as the transarterial approach detailed here, we assert that the most appropriate implantation procedure should be chosen based on patient background, target location, anatomical features, and the skills available at each institution.

According to previous reports, the median OS of pancreatic cancer treated by Cyberknife ranges from 10.6 to 18.6 months, 1-year OS ranges from 39.1 to 56%, and PFS ranges from 7.3 to 9.8 months [11–16]. Our reported median OS and 1-year OS results were within the range of previous reports and PFS was a little bit higher. The RT dose of our study (45–60 Gy/5–15 Fr) tended to be higher than other reports, which could explain the clinical outcome, limiting the conclusions that can be drawn from this study on the success of this approach. Further, this study was limited by its nonrandomized patient inclusion, because it was a single institution retrospective study.

In conclusion, we performed transarterial fiducial marker implantation for real-time tracking of radiotherapy in 14 pancreatic cancer patients without complication, and this approach may be an alternative method for fiducial marker implantation for real-time tracking of irradiation when other approaches are difficult to perform.

## References

[1] Chang B, Timmerman R (2007). Stereotactic body radiation therapy. Am J Clin Oncol.

[2] Song Y, Yuan Z, Li F, Dong Y, Zhuang H, Wang J (2015). Analysis of clinical efficacy of CyberKnife treatment for locally advanced pancreatic cancer. Onco Targets Ther.

[3] Bhasin DK, Rana SS, Jahagirdar S, Nagi B (2006). Does the pancreas move with respiration?. J Gastroenterol Hepatol.

[4] Nishita K, Jeremy JH, John DL, William TK, Billy WL, Albert K (2009). Safety and efficacy of percutaneous fiducial marker implantation for image-guided radiation therapy. J Vasc Interv Radiol.

[5] Cristoph GT, Sophia MH, Alexander M, Robert S, Sebastian S, Philipp MP (2014). CT fluoroscopy–guided percutaneous fiducial marker placement for CyberKnife stereotactic radiosurgery: technical results and complications in 222 consecutive procedures. J Vasc Interv Radiol.

[6] Kanda Y (2013). Investigation of the freely-available easy-to-use software “EZR” (Easy R) for medical statistics. Bone Marrow Transplant.

[7] Bertelli E, Di Gregorio F, Bertelli L, Mosca S (1995). The arterial blood supply of the pancreas: a review I. The superior pancreaticoduodenal and the anterior superior pancreaticoduodenal arteries. An anatomical and radiological study. Surg Radiol Anat.

[8] Aline CP, Brian C, Gregory G, Sushil A, Nadim GH (2006). EUS-guided fiducial placement for CyberKnife radiotherapy of mediastinal and abdominal malignancies. Gastrointest Endosc.

[9] Walter GP, Brian MY, Devin S, Jeff K, Daniel TC, Albert K (2010). EUS-guided gold fiducial insertion for image-guided radiation therapy of pancreatic cancer: 50 successful cases without fluoroscopy. Gastrointest Endosc.

[10] Sanders MK, Moser AJ, Khalid A, Fasanella KE, Zeh HJ, Burton S (2010). EUS-guided fiducial placement for stereotactic body radiotherapy in locally advanced and recurrent pancreatic cancer. Gastrointest Endosc.

[11] Polistina F, Costantin G, Casamassima F, Francescon P, Guglielmi R, Panizzoni G (2010). Unresectable locally advanced pancreatic cancer: a multimodal treatment using neoadjuvant chemoradiotherapy (gemcitabine plus stereotactic radiosurgery) and subsequent surgical exploration. Ann Surg Oncol.

[12] Rwigema JC, Parikh SD, Heron DE, Howell M, Zeh H, Moser AJ (2011). Stereotactic body radiotherapy in the treatment of advanced adenocarcinoma of the pancreas. Am J Clin Oncol.

[13] Gurka MK, Collins SP, Slack R, Tse G, Charabaty A, Ley L (2013). Stereotactic body radiation therapy with concurrent full-dose gemcitabine for locally advanced pancreatic cancer: a pilot trial demonstrating safety. Radiat Oncol.

[14] Didolkar MS, Coleman CW, Brenner MJ, Chu KU, Olexa N, Stanwyck E (2010). Image-guided stereotactic radiosurgery for locally advanced pancreatic adenocarcinoma results of first 85 patients. J Gastrointest Surg.

[15] Goyal K, Einstein D, Ibarra RA, Yao M, Kunos C, Ellis R (2012). Stereotactic body radiation therapy for nonresectable tumors of the pancreas. J Surg Res.

[16] Moningi S, Dholakia AS, Raman SP, Blackford A, Cameron JL, Le DT (2015). The role of stereotactic body radiation therapy for pancreatic cancer: a single-institution experience. Oncology.

