# Correction to: Transarterial fiducial marker implantation for CyberKnife radiotherapy to treat pancreatic cancer: an experience with 14 cases

**DOI:** 10.1007/s11604-023-01405-2

**Published:** 2023-03-09

**Authors:** Akira Imaizumi, Takuji Araki, Hiroki Okada, Yu Sasaki, Takafumi Komiyama, Toshihiro Suzuki, Hiroshi Takahashi, Hiroshi Onishi

**Affiliations:** 1 Department of Radiology, Yamanashi University, 1110 Shimokato, Chuo, Yamanashi 409-3898 Japan; 2 Kasugai CyberKnife Rehabilitation Hospital, 436 Kokufu, Kasugai-Cho, Fuefuki, Yamanashi 406-0014 Japan

**Correction to: Japanese Journal of Radiology (2021) 39:84–92** 10.1007/s11604-020-01040-1

In the original publication, the last sentence under heading Patients in section Materials and methods should read as:

All patients gave written informed consent for the procedure.

The revised Ethical approval statement should read as:

**Ethical approval** All procedures performed in these studies involving human participants were in accordance with the ethical standards of the institutional and/or national research committee and with the 1964 Helsinki Declaration and its later amendments. All patients gave written informed consent for the procedure. The institutional review board of our hospital approved retrospective data analysis.

